# Erratum to “BRD2 induces drug resistance through activation of the RasGRP1/Ras/ERK signaling pathway in adult T-cell lymphoblastic lymphoma”

**DOI:** 10.34133/cancomm.0061

**Published:** 2026-11-03

**Authors:** Xiao-Peng Tian, Jun Cai, Shu-Yun Ma, Yu Fang, Hui-Qiang Huang, Tong-Yu Lin, Hui-Lan Rao, Mei Li, Zhong-Jun Xia, Tie-Bang Kang, Dan Xie, Qing-Qing Cai

**Affiliations:** ^1^ State Key Laboratory of Oncology in South China, Collaborative Innovation Center for Cancer Medicine, Sun Yat-sen University Cancer Center, Guangzhou, Guangdong 510060, P. R. China.; ^2^ Department of Medical Oncology, Sun Yat-sen University Cancer Center, Guangzhou, Guangdong 510060, P. R. China.; ^3^ Department of Pathology, Sun Yat-sen University Cancer Center, Guangzhou, Guangdong 510060, P. R. China.; ^4^ Department of Hematology, Sun Yat-sen University Cancer Center, Guangzhou, Guangdong 510060, P. R. China.

Following publication of this article, the authors noticed that the WB bands of c-Myc in Figure 7D were inadvertently misplaced [1]. After carefully checking the original data, the authors confirmed the error occurred during figure assembly. This error does not affect the conclusions of this article. The figure has been corrected in the original HTML and PDF, and Fig. 7D is provided below. The authors would like to apologize for any inconvenience caused.

**Fig. 7.**
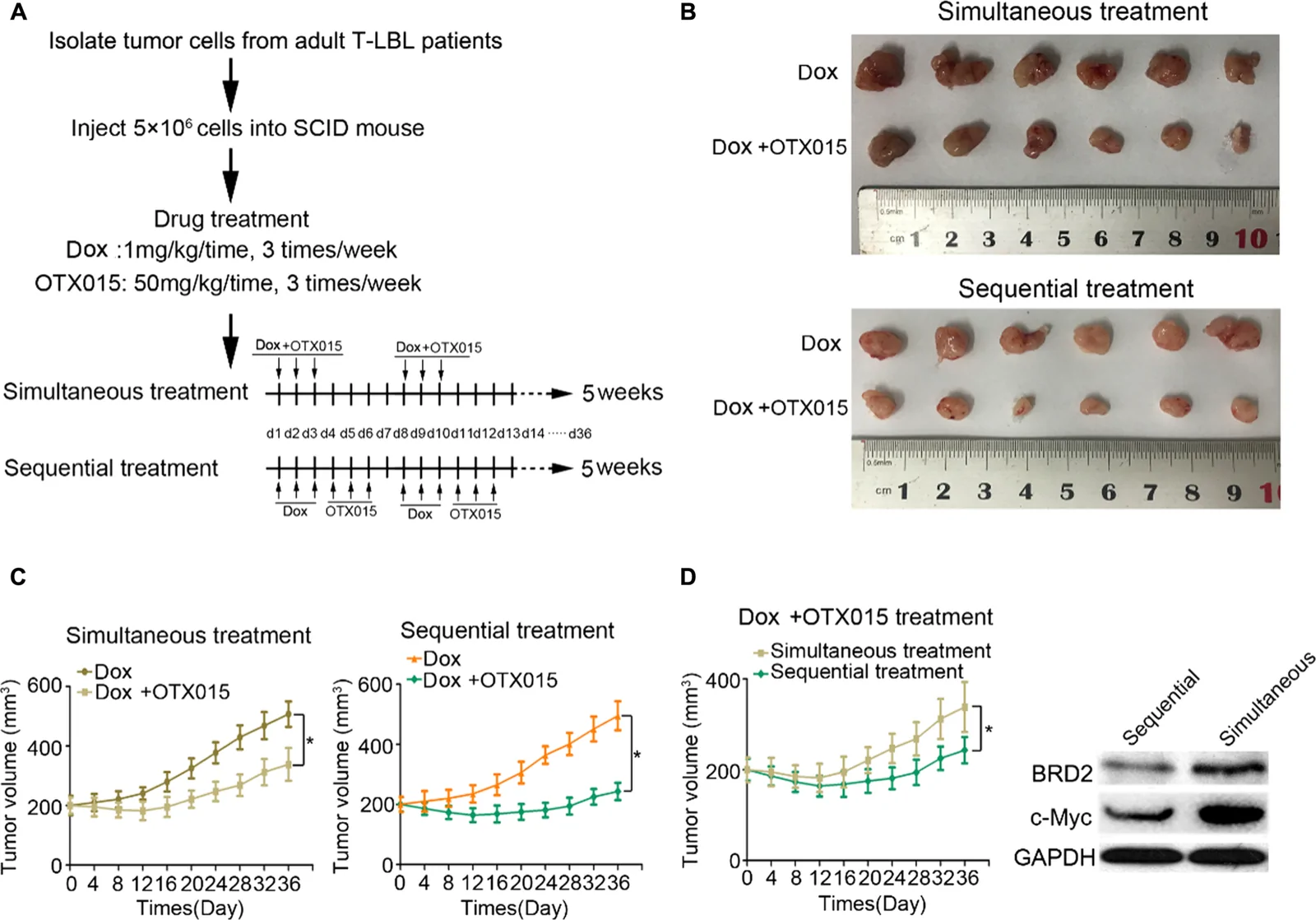
The comparison of simultaneous and sequential treatment of Dox combined with OTX015 using a PDX model of T-LBL. A. The flowchart of simultaneous and sequential treatment of Dox combined with OTX015 on PDX model. B. PDXs subjected to sequential treatment were smaller at day 36 than those subjected to simultaneous treatment. C. Tumor size of xenograft was measured every 4 days for 36 days. The mean tumor volume in the drug combination group (Dox + OTX015) was significantly smaller than that of the Dox group. D. The mean tumor volume of the simultaneous treatment group was significantly larger than that of the sequential treatment group (left panel). Right: The protein expression levels of BRD2 and c-Myc were detected by Western blotting in xenografts. *, *P* < 0.05. Abbreviations: T-LBL, T-cell lymphoblastic lymphoma; Dox, doxorubicin; PTX, patient-derived xenograft

## References

[1] Tian X-P, Cai J, Ma S-Y, Fang Y, Huang H-Q, Liu T-Y, Rao H-L, Li M, Xia Z-J, Kang T-B, et al. BRD2 induces drug resistance through activation of the RasGRP1/Ras/ERK signaling pathway in adult T-cell lymphoblastic lymphoma. Cancer Commun. 2020;40(6):245–259. 10.1002/cac2.12039 PMC7307265 32459053

